# Immunohistological features related to functional impairment in lymphangioleiomyomatosis

**DOI:** 10.1186/s12931-018-0797-9

**Published:** 2018-05-08

**Authors:** Ellen Caroline Toledo do Nascimento, Bruno Guedes Baldi, Alessandro Wasum Mariani, Raquel Annoni, Ronaldo Adib Kairalla, Suzana Pinheiro Pimenta, Luiz Fernando Ferraz da Silva, Carlos Roberto Ribeiro Carvalho, Marisa Dolhnikoff

**Affiliations:** 1 Departamento de Patologia, Faculdade de Medicina FMUSP, Universidade de Sao Paulo, Av. Dr. Arnaldo, 455, room 1155, Cerqueira Cesar, CEP 01246-903 Sao Paulo, SP Brazil; 2 Divisao de Pneumologia, Instituto do Coracao, Hospital das Clinicas HCFMUSP, Faculdade de Medicina FMUSP, Universidade de Sao Paulo, Sao Paulo, SP Brazil; 3 Departamento de Cirurgia Toracica, Instituto do Coracao, Hospital das Clinicas HCFMUSP, Faculdade de Medicina FMUSP, Universidade de Sao Paulo, Sao Paulo, SP Brazil

**Keywords:** Lymphangioleiomyomatosis, Pulmonary function tests, VEGF-D, Metalloproteinase, mTOR, Immunohistochemistry

## Abstract

**Background:**

Lymphangioleiomyomatosis (LAM) is a low-grade neoplasm characterized by the pulmonary infiltration of smooth muscle-like cells (LAM cells) and cystic destruction. Patients usually present with airway obstruction in pulmonary function tests (PFTs). Previous studies have shown correlations among histological parameters, lung function abnormalities and prognosis in LAM. We investigated the lung tissue expression of proteins related to the mTOR pathway, angiogenesis and enzymatic activity and its correlation with functional parameters in LAM patients.

**Methods:**

We analyzed morphological and functional parameters of thirty-three patients. Two groups of disease severity were identified according to FEV1 values. Lung tissue from open biopsies or lung transplants was immunostained for SMA, HMB-45, mTOR, VEGF-D, MMP-9 and D2-40. Density of cysts, density of nodules and protein expression were measured by image analysis and correlated with PFT parameters.

**Results:**

There was no difference in the expression of D2-40 between the more severe and the less severe groups. All other immunohistological parameters showed significantly higher values in the more severe group (*p* ≤ 0.002). The expression of VEGF-D, MMP-9 and mTOR in LAM cells was associated with the density of both cysts and nodules. The density of cysts and nodules as well as the expression of MMP-9 and VEGF-D were associated with the impairment of PFT parameters.

**Conclusions:**

Severe LAM represents an active phase of the disease with high expression of VEGF-D, mTOR, and MMP-9, as well as LAM cell infiltration. Our findings suggest that the tissue expression levels of VEGF-D and MMP-9 are important parameters associated with the loss of pulmonary function and could be considered as potential severity markers in open lung biopsies of LAM patients.

## Background

Lymphangioleiomyomatosis (LAM) is a rare low-grade neoplasm that affects women of reproductive age, characterized by the proliferation of immature smooth muscle-like cells (LAM cells), mainly in the lungs [1–3]. LAM often arises spontaneously in patients with no evidence of genetic disease and is present in approximately one-third of women with tuberous sclerosis complex (TSC) [4]. The main clinical features in LAM patients include progressive dyspnea and recurrent pneumothorax, whereas the main findings in pulmonary function tests (PFTs) are an obstructive pattern and reduced diffusion capacity for carbon monoxide (DL_CO_). The clinical course is variable; some patients have slow progression over many years, while others develop progressive respiratory failure a few years after disease onset [5, 6].

The pathologic changes in pulmonary LAM (LAM lesions) are characterized by two distinct components: the cystic and the cellular components [7]. The former consists of multiple cysts distributed diffusely throughout the lungs and the latter is composed by two cellular subpopulations (LAM cells): spindle myofibroblast-like cells and polygonal cells with epithelioid morphology. LAM cells predominantly form nodules, mainly localized at the cysts walls, but small cell clusters can also be found scattered in the lung parenchyma [8]. The spindle cell population expresses smooth muscle proteins and forms the core of the nodules, surrounded by epithelioid cells that exhibit immunoreactivity for theHMB-45 antibody, a typical melanocytic cell marker [9]. The cell proliferation has been proposed to play a central role in the destruction of lung parenchyma due to high proliferative activity, growth factor release and metalloproteinase (MMP) production [10]. Moreover, the dysregulation and hyperactivation of the mTOR kinase pathway leads to increased protein translation and ultimately to inappropriate cellular proliferation, migration and invasion [11, 12].

Previous studies have shown the correlation of morphological parameters with prognosis determined by overall survival. Matsui et al. (2001) showed that certain histological criteria, such as the percentage of lung tissue cysts, the degree of cell infiltration into the tissue and the accumulation of hemosiderin-laden macrophages, correlate with worse prognosis [7]. Kitaichi et al. (1995) also suggested that higher grades of predominantly cystic lesions correlated inversely with survival from 2 to 5 years after biopsy. However, the previous studies that evaluated the histological parameters related to prognosis mainly focused on survival; the morphological determinants of impairment of PFTs have yet to be identified [13].

To investigate the association of immunohistological features with impairment in PFTs, we evaluated morphological parameters related to tissue destruction, LAM cell infiltration, and the expression of proteins related to the mTOR pathway, angiogenesis and enzymatic activity in LAM patients with different functional severities.

## Methods

The study was approved by the review board for the human ethics committee of Sao Paulo University (CAPPesq-FMUSP n° 0623/09). The study is retrospective and used medical records and archived material from the Department of Pathology of Sao Paulo University Medical School.

### Study population

Lung tissue from thirty-three patients with LAM who underwent diagnostic open lung biopsies or lung transplants between 2003 and 2011 was retrospectively included in the study. The diagnosis of LAM was reviewed and established according to the guidelines of the European Respiratory Society [6].

### Clinical features, pulmonary function tests and serum levels of VEGF-D

The following clinical features were evaluated: age at surgical procedure; age at diagnosis; presence of TSC; smoking history; pulmonary and extrapulmonary manifestations.

All patients underwent spirometry, determination of lung volumes, and determination of DL_CO_using a whole-body plethysmograph (Elite D MedGraphics; Medical Graphics Co., Saint Paul, MN, USA). Pulmonary function testing was performed in accordance with the recommended guidelines for pulmonary function testing in Brazilian adults. The reference values for spirometry, lung volumes, and DL_CO_ were the ones established for the Brazilian population [14–16].

According to functional parameters at the time of lung biopsy or prior to lung transplantation, we identified two groups of patients based on disease severity: 1) the less severe group (*n* = 19), which included patients with FEV_1_ ≥ 75% of the predicted value; and 2) the more severe group (*n* = 12), which included patients with FEV_1_ ≤ 51% of the predicted value. Two patients presented borderline predicted values of FEV_1_ (67 and 68%).

Serum levels of VEGF-D were measured using a commercially available enzyme-linked immunosorbent assay kits (Quantikine Human VEGF-D Immnoassay) according to the manufacturer’s instruction (ELISA; R&D Systems, Minneapolis, MN, USA). The detection limit of the assay was 15.6 pg/mL.

### Tissue processing and histological analysis

Paraffin blocks of lung tissue were retrieved from the archives of the Department of Pathology of Sao Paulo University Medical School. The tissue had been previously fixed in 10% buffered formalin for 24 h, routinely processed and embedded in paraffin. Serial sections 5 μm thick were stained with hematoxylin and eosin (H&E) and subjected to immunohistochemistry.

H&E stained biopsy sections were evaluated by two pathologists (ECTN and MD). One slide or two slides per patient, depending on tissue availability, were examined after immunohistochemical staining, which was performed as previously described [17, 18]. Briefly, sections were deparaffinized, and a 0.3% hydrogen peroxide solution was applied for 35 min to inhibit endogenous peroxidase activity. Antigen retrieval was performed for 45 min using a citrate solution. Sections were incubated with the primary antibody overnight at 4 °C using the following antibodies: SMA, clone 1A4, 1:1000, Dako, California, United States; HMB-45, clone HMB-45, 1:400, Dako, California, United States; VEGF-D, clone 78,923, 1:7000, R&D Systems, United Kingdom.; D2-40, clone D2-40, 1:1000, Dako, California, United States; mTOR, clone 7C10, 1:180, Abcam, United Kingdom; MMP-9, clone 15 W2, 1:350, Novocastra, United Kingdom. Novolink Polymer (Novocastra) was used as the secondary antibody, and 3, 3′-diaminobenzidine (Sigma Chemical Co, St Louis, Mo) was used as the chromogen. The sections were counterstained with Harris hematoxylin. For negative controls, the primary antibody was replaced by a competing peptide used to raise the antibody.

**Fig. 1:**
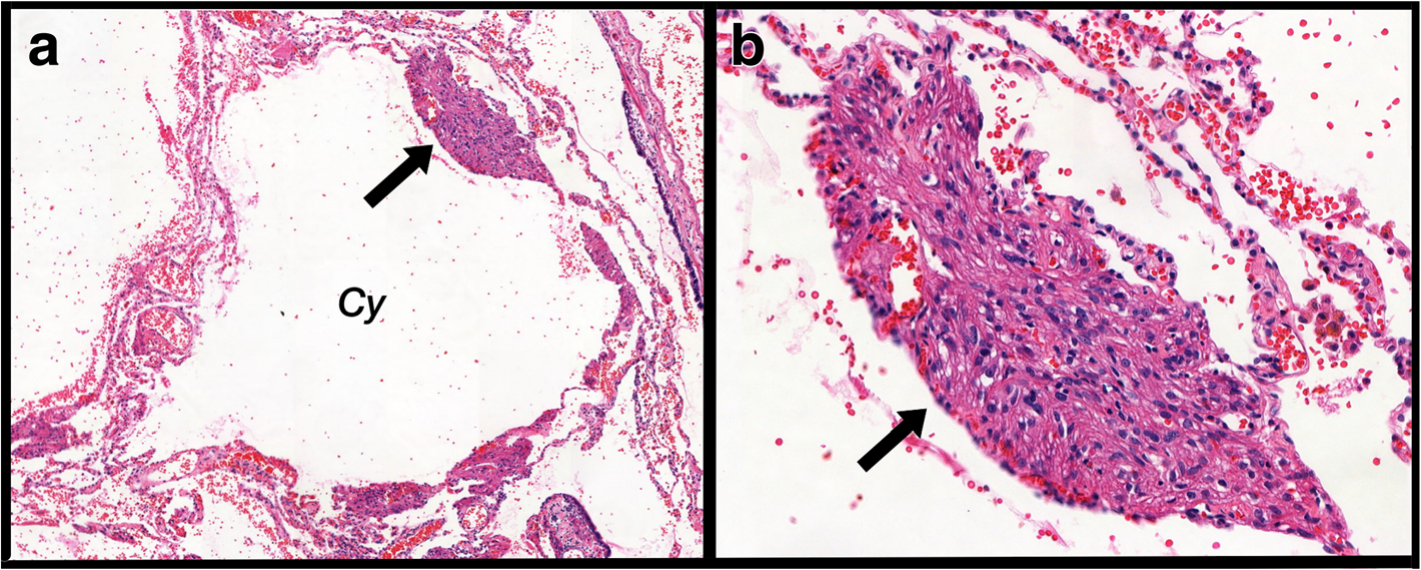
Representative LAM lesion stained at H&E (**a**). Panel **b** shows a higher magnification of the typical LAM cells nodule. Cy = cystic component; arrow indicates the cellular component (LAM cells nodule) localized at the cyst wall. Magnifications: A = 70X, B = 200X

**Fig. 2:**
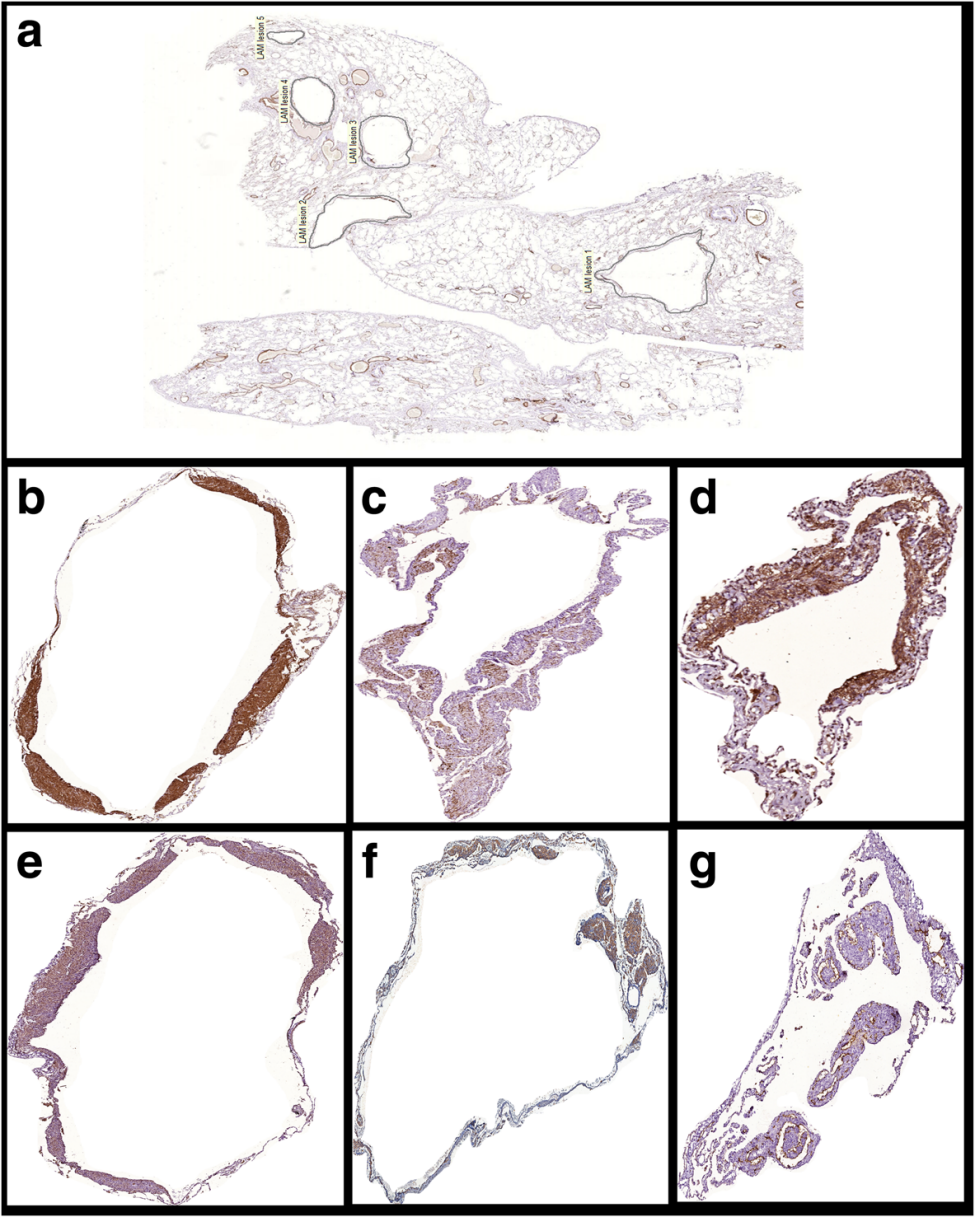
Representative photomicrographs of LAM lesions extracted for image analysis. Panel **a** shows an SMA-stained lung biopsy with multiple LAM lesions. Panels **b** to **g** show examples of extracted lesions from different immunohistochemical stains. **b** - SMA, **c** - HMB45, **d** - MMP9, **e** - VEGFD, **f** - mTOR, **g** - D2-40. Magnifications: A = 10X, B to G = 40X

**Fig. 3:**
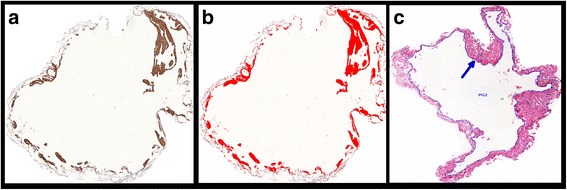
Representative image analysis. Panel **a** shows an SMA-stained extracted LAM lesion. Panel **b** shows the positively stained area (red) at image analysis. Panel **c** shows the internal perimeter (arrow pointing to the blue perimeter line) of the cystic component of a LAM lesion (H&E staining). Magnification: 40X

For image analysis (using Image-Pro Plus 4.1 for Windows image analysis software [Media Cybernetics, Silver Spring, MD, USA]), the whole tissue area within the slide was included in the measurements. All the slides were digitized using a Panoramic Viewer® software, version 1.15.2 for Windows® (3DHistech, Budapest, Hungary). All LAM lesions, consisting of cysts and nodules, were individualized and extracted in each slide. Figures 1 and 2 show, respectively, a representative LAM lesion stained at H&E and LAM lesions extracted for image analysis. During image analysis, the following parameters were assessed: a) lung tissue area: calculated as the total area of the biopsy minus the area occupied by air. The total area varies among the cases because it is related to the size of the biopsy and the degree of tissue insufflation. Therefore, protein expression was normalized for the lung tissue area so as to avoid biases related to the sample size and variation in the lung insufflation; b) area of cysts: assessed using the internal perimeter of each cystic component of LAM lesions (Fig. 3c). The index of cystic tissue destruction (density of cysts) was calculated as the sum of cystic areas, corrected for the lung tissue area; c) positivity of smooth muscle actin (SMA), HMB-45, vascular endothelial growth factor-D (VEGF-D), podoplanin (D2-40), mammalian target of rapamycin (mTOR), and matrix metalloproteinase-9 (MMP-9): assessed as the positively stained areas in each LAM lesion (Fig. 3a and b). Positivity for each antibody staining was determined by using the color threshold. For this purpose, different sections stained with each antibody were used to obtain the best range of positivity in the cases, which were examined by 2 pathologists (ECTN and LFFS). These procedures generated a file that contained all of the color selection data, which were subsequently applied to all cases stained with the same antibody. The expression levels of VEGF-D, D2-40, mTOR, and MMP-9 were calculated as the sum of positively stained areas in the LAM lesions, corrected for the lung tissue area; d) the index of LAM cell infiltration (density of nodules) was calculated as the sum of SMA-positive and HMB45-positive areas, corrected for the lung tissue area.

### Statistical analysis

Data are presented as the mean ± SD, as the median (interquartile range) or as numbers (percentile). The Mann-Whitney *U* test was used to compare the immunohistological and functional data between the two groups. Categorical variables were compared with Fisher exact test. The correlation between immunohistological and functional data (FEV_1_ and DL_CO_) in the 33 patients was analyzed by the Spearman rank test. Time-to-event (death or lung transplantation) analysis was performed with Kaplan-Meier method and differences between groups were evaluated with Log-Rank tests. The level of significance was set at *p* < 0.05. Statistical analysis was performed using the statistical software SPSS 15.0 (SPSS, Chicago, Ill).

## Results

Thirty-three women with LAM were included in the study, 7 (21%) of whom underwent lung transplantation while 26 (79%) underwent lung biopsy for diagnostic purposes. The clinical features are described in Table 1. The mean age at pulmonary biopsy or transplantation was 42 ± 8 years, 3 (9%) had underlying TSC, and 15 (46%) had renal angiomyolipoma. The most common symptom was dyspnea (70%), and 22 (67%) patients had a history of pneumothorax.

**Table 1:** Clinical features (*n* = 33)

Age at pulmonary biopsy or transplantation, years	42 ± 8
Age at diagnosis, years	40 ± 8
Presence of TSC	3 (9%)
Renal angiomyolipoma	15 (46%)
Ex-smokers	7 (21%)
Dyspnea	23 (70%)
Baseline dyspnea index	10 (8 - 12)
Pneumothorax	22 (67%)
Hemoptysis	5 (15%)
Cough	4 (12%)
Chylothorax	3 (9%)
Supplemental oxygen	6 (18%)

Values are the mean ± SD, median (interquartile range) or percentage (%)

*TSC* tuberous sclerosis complex

Pulmonary function tests are described in Table 2. The mean FEV_1_ and DL_CO_ were 2.05 ± 0.93 L (72 ± 31% of predicted) and 17.4 ± 7.6 ml/min/mmHg (67 ± 29% of predicted), respectively.

**Table 2:** Pulmonary function tests (*n* = 33)

FEV_1_, L	2.05 ± 0.93
%predicted	72 ± 31
FVC, L	3.05 ± 0.92
%predicted	89 ± 24
FEV_1_/FVC	0.64 ± 0.19
RV, L	2.04 ± 0.81
%predicted	138 ± 53
TLC, L	5.19 ± 0.83
%predicted	105 ± 14
RV/TLC	0.36 ± 0.13
DL_CO_, ml/min/mmHg	17.4 ± 7.6
%predicted	67 ± 29

Values are mean ± SD

*DL*_*CO*_ lung diffusion capacity for carbon monoxide, *FEV*_*1*_ forced expiratory volume in the first second, *FVC* forced vital capacity, *RV* residual volume, *TLC* total lung capacity

The histological features were consistent with those previously reported [7, 12, 13]. There was significant variability in the number of LAM lesions among the cases, with extensive lung involvement in the more severe group. The immunohistochemical evaluation showed cytoplasmatic localization for each antibody. All analyzed proteins were expressed in the LAM cells. SMA was also expressed in the normal smooth muscle fibers of the airway and vessel walls; D240 in the lymphatic vessels; MMP9 in the macrophages, and VEGF-D in the areas of capillary proliferations, mainly within the pleural surface.

**Fig. 4:**
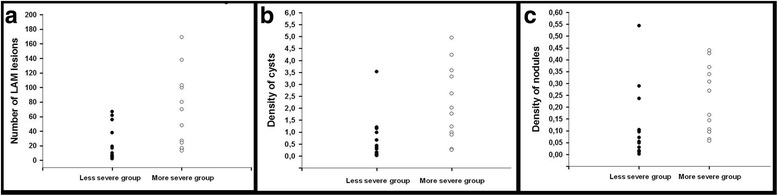
The graphs show the variability in the number of LAM lesions (**a**), density of cysts (**b**), and density of nodules (**c**) among cases in the two groups

**Fig. 5:**
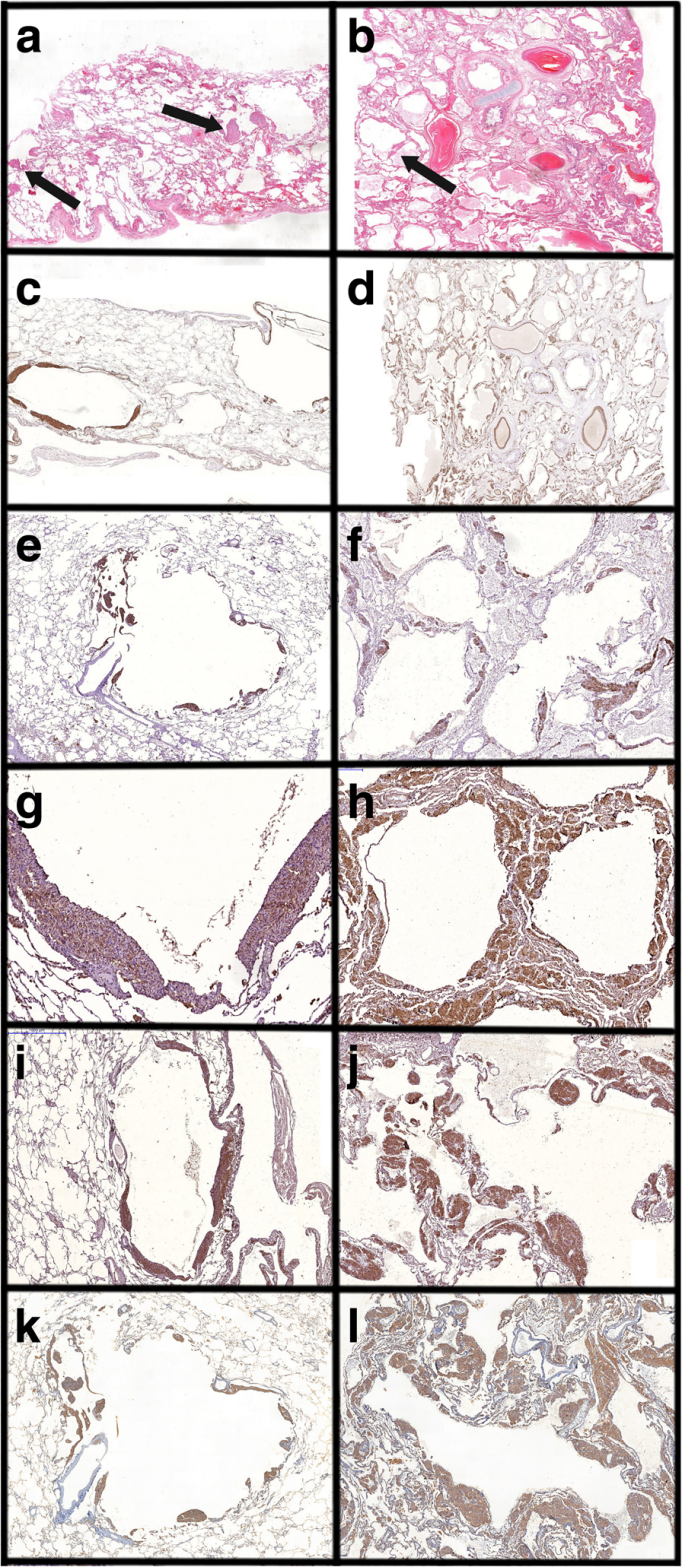
Representative histological images of H&E staining and immunohistochemistry for protein expression (stained in brown) in each group. All immunohistological parameters showed significantly higher values in the more severe group. **a**, **c**, **e**, **g**, **i**, and **k**: the less severe group; **b**, **d**, **f**, **h**, **j**, and **l**: the more severe group. A and B: H&E staining showing the distribution of cysts and LAM-cells nodules (arrows) in each group (magnification: 5×). **c** and **d**: SMA expression in LAM-cells (magnification: C, 7× and D, 7×). **e** and **f**: HMB-45 expression in LAM-cells (magnification: E, 20× and F, 40×). **g** and **h**: VEGF-D expression in LAM-cells (magnification: G, 100× and H, 100×). **i** and **j**: MMP-9 expression in LAM-cells (magnification: I, 25× and J, 25×). **k** and **l**: mTOR expression in LAM-cells (magnification: K, 35× and L, 35×)

Table 3 compares the morphological, laboratorial, functional and survival/lung transplantation data between the two groups (less severe and more severe). Two patients presented borderline predicted values of FEV_1_ (67 and 68%) and were not included in this comparative analysis. There was no difference in the expression of D2-40 between the more severe and the less severe groups. All other immunohistological parameters showed significantly higher values in the more severe group (*p* ≤ 0.002). Serum levels of VEGF-D were available for 23 patients; there was no difference between the less severe and the more severe groups. Figure 4 shows the variability in the number of LAM lesions, density of cysts, and density of nodules among cases in the two groups. Representative photomicrographs of immunohistological changes in both groups are shown in Fig. 5.The correlations among immunohistological parameters are shown in Table 4. The expression levels of VEGF-D, MMP-9 and mTOR in LAM cells were associated with both the density of cysts and the density of nodules. Further, the density of cysts showed a positive correlation with the density of nodules (*r* = 0.62; *p* < 0.001).

**Table 3:** Comparison of morphological, laboratorial, functional, and survival/lung transplantation data between the two groups

Parameters	Less severe (FEV_1_ ≥ 75% of predicted); *n* = 19	More severe (FEV_1_ ≤ 51% of predicted); *n* = 12	*p*
Density of nodules	0.048 (0.09)	0.218 (0.26)	0.002
Density of cysts	0.337 (0.93)	1.903 (2.61)	0.001
LAM lesions/case	10 (19)	59 (84)	0.001
D2-40	0.001 (0.010)	0.008 (0.004)	0.087
MMP-9	0.011 (0.024)	0.066 (0.096)	< 0.001
mTOR	0.001 (0.010)	0.013 (0.024)	0.002
VEGF-D	0.010 (0.019)	0.058 (0.067)	0.002
serum VEGF-D^a^ (pg/mL)	821 (1273)	1411 (1468)	0.15
FEV_1_ (L)	2.73 ± 0.49	1.01 ± 0.41	< 0.001
FEV_1_ (% predicted)	95.05 ± 14.01	35.33 ± 12.60	< 0.001
DLco (ml/min/mmHg)	21.00 ± 4.95	8.09 ± 2.67	< 0.001
DL_CO_ (% predicted)	80.26 ± 20.59	33.89 ± 11.49	< 0.001
Death *n* (%)	0	2 (16.6)	
Lung transplantation *n* (%)	0	7 (58.3)	
Referred to lung transplantation *n* (%)	0	3 (25)	

Values are median (interquartile range) or mean ± SD or number of cases (%)

*DL*_*CO*_ diffusion capacity for carbon monoxide, *D2-40* podoplanin, *FEV*_*1*_ forced expiratory volume in the first second, *MMP-9* metalloproteinase 9, *mTOR* mammalian target of rapamycin, *VEGF-D* vascular endothelial growth factor-D

Density of nodules is expressed as the relation between area of nodules and lung tissue area. Density of cysts is expressed as the relation between the area of cysts and lung tissue area. Protein expression is presented as the relation between area of positive staining and lung tissue area

^a^Serum levels of VEGF-D were available for 17 patients in the less severe group and 6 patients in the more severe group

**Table 4:** Correlations among immunohistological parameters (*n* = 33)

	VEGF-D	MMP-9	mTOR	D2-40	Density of cysts
Density of cysts	*r* = 0.67 *p* < 0.001	*r* = 0.70 *p* < 0.001	*r* = 0.67 *p* < 0.001	ns	–
Density of nodules	*r* = 0.64 *p* < 0.001	*r* = 0.76 *p* < 0.001	*r* = 0.54 *p* = 0.003	*r* = 0.66 *p* < 0.001	*r* = 0.62 *p* < 0.001

*ns* not significant, *D2-40* podoplanin, *MMP-9* metalloproteinase 9, *mTOR* mammalian target of rapamycin, *VEGF-D* vascular endothelial growth factor-D

Table 5 presents the correlations between immunohistological findings and functional parameters. The density of cysts, the density of nodules and the expression of MMP-9 and VEGF-D were associated with impairment in PFTs (FEV_1_ and DL_CO_).

**Table 5:** Correlations between immunohistological findings and lung function parameters (*n* = 33)

	Density of nodules	Density of cysts	VEGF-D	MMP-9	mTOR	D2-40
FEV_1_ (%predicted)	*r* = −0.51 *p* = 0.003	*r* = −0.58 *p* < 0.001	*r* = − 0.60 *p* < 0.001	*r* = − 0.62 *p* < 0.001	*r* = − 0.55 *p* = 0.002	ns
DLco (%predicted)	*r* = − 0.68 *p* < 0.001	*r* = − 0.53 *p* = 0.003	*r* = − 0.51 *p* = 0.004	*r* = − 0.66 *p* < 0.001	ns	*r* = − 0.48 *p* = 0.008

*ns* not significant, *DL*_*CO*_ diffusion capacity for carbon monoxide, *D2-40* podoplanin, *FEV*_*1*_ forced expiratory volume in the first second, *MMP-9* metalloproteinase 9, *mTOR* mammalian target of rapamycin, *VEGF-D* vascular endothelial growth factor-D

**Fig. 6:**
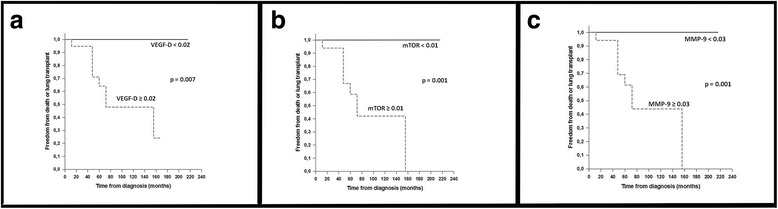
Kaplan-Meier estimates of freedom from death or lung transplantation for tissue expression of VEGF-D (**a**), mTOR (**b**), and MMP-9 (**c**). For each marker, two subgroups of patients were defined according to the marker median values

Figure 6 shows the Kaplan-Meier survival curves for tissue expression levels of VEGF-D, mTOR, and MMP-9. For each marker, two subgroups of patients were defined according to the marker median values. In the subgroup with lower median values, no patient died or underwent lung transplantation, whereas in the subgroup with higher median values 47% (VEGF-D), 53% (MMP-9) or 56% (mTOR) of the patients died or underwent lung transplantation (*p* ≤ 0.007). The median time to death or lung transplantation was 72 months.

## Discussion

In this study examining open lung biopsy specimens and lung explants from patients with LAM, we analyzed a range of histological and immunohistochemical markers that represent parameters of tissue destruction, lymphangiogenesis, LAM-cells nodules and mTOR-pathway alterations. Our results showed that 1) the more severe, advanced phase of disease presents with increased mTOR, VEGF-D and MMP-9 expression, as well as greater extent of cystic areas and LAM cell nodules; and 2) the main immunohistochemical and histological parameters associated with loss of pulmonary function were MMP-9, VEGF-D, the density of cysts and the density of nodules. These results provide new insights into the natural history of the disease and show for the first time that the tissue expression of VEGF-D in LAM cells is associated with cystic tissue destruction, LAM cell infiltration and loss of pulmonary function.

Lymphangioleiomyomatosis (LAM) is a rare disease of unknown etiology characterized by an abnormal infiltration of cells expressing smooth muscle protein in the lungs, lymph nodes or other locations, and often associated with renal angiomyolipoma [19, 20]. The disorder was previously classified as an interstitial lung disease and tumor-like lesion but is now considered a low-grade malignancy, with destructive metastasizing potential and evidence of clonal origin, being part of the spectrum of PEComatous tumors [1, 2, 21]. It has a variable course but may lead to progressive lung cystic destruction, respiratory failure and terminal lung disease [21].

Previous studies have shown that quantitative computed tomography (CT) can be used to evaluate the severity of lung disease in LAM patients. The quantitative CT scan grading has a good correlation with lung function tests, exercise performance and gas exchange [22]. Several authors have demonstrated a correlation between the extent of cysts on CT and the degree of airflow obstruction and reduced DLCO levels [23–25]. In consonance with CT studies, we used a quantitative histological method to assess cystic tissue destruction and also demonstrated that the results are associated with loss of pulmonary function, reductions in both FEV_1_ and DL_CO_, which are considered important prognostic markers in LAM. Semiquantitative histologic studies already demonstrated that the percentage of predominantly cystic lesions is associated with prognosis. Kitaichi et al. (1995) used an individual score for each component of LAM lesions, that is, cysts and muscular-type cell infiltration, to demonstrate that predominantly cystic lesions have a tendency toward poor prognosis compared with predominantly muscular-type lesions [13]. The LAM histologic score established by Matsui et al. (2001), based on the total percentage of lung tissue involvement by both the smooth muscle-type cell and the cystic components, showed a strong correlation with the overall survival of LAM patients [7]. Both studies have an important aspect in common: the percentage of cystic component alone was a predictor of survival, independently of the extent of LAM cell infiltration. In this study, we used quantitative image-analysis methods to measure LAM lesion components individually (cysts and nodules), adding new elements to previous histological studies on LAM. Our results showed that both tissue destruction and LAM cell infiltration, assessed respectively by the density of cysts and the extent of LAM cell nodules, are associated with loss of pulmonary function. The stronger association of cystic areas with lower values of FEV_1_ indicates that cystic tissue destruction is likely to be more relevant than cell infiltration in disease progression, as previously suggested. However, patients with more severe disease presented an increased extent of LAM cell nodules with higher expression of MMP9, VEGF-D and mTOR, suggesting that rather than a quiescent predominantly cystic phase, LAM cells present high metabolic activity in the advanced phases of the disease, likely participating in the process of tissue destruction.

Metalloproteinases are involved in both cystic destruction and the metastatic potential associated with pulmonary LAM cells [26]. The pathogenesis of cystic destruction of lung parenchyma seems to be associated with an imbalance between MMPs and tissue inhibitor of MMPs, with increased levels of MMPs [10]. Our results are consistent with this concept, showing a strong association of MMP expression with the extent of cystic areas and also with functional impairment.

Vascular endothelial growth factor (VEGF) is a major angiogenic growth factor produced by malignant cells. VEGF-D, a ligand for the lymphatic growth-factor receptor VEGFR-3/Flt-4, induces the formation of lymphatics and promotes the spread of tumor cells to the lymph nodes [27, 28]. Seyama et al. reported that levels of VEGF-D, but not VEGF-A or VEGF-C, are elevated in patients with sporadic lymphangioleiomyomatosis compared with healthy controls [29]. It has been suggested that the serum level of VEGF-D is a valuable marker of LAM diagnosis and severity and to evaluate the response to treatment [30–32]. VEGF-D is upregulated through the mTOR signaling pathway, is expressed by LAM cells, and is associated with LAM cell proliferation, dissemination and lymphangiogenesis [28–30, 33]. Previous studies have assessed the tissue expression of VEGF-C by LAM cells as a marker of lymphangiogenesis, which correlated with the LAM histologic score [19]. Xu et al. (2013) and Baldi et al. (2014) reported that levels of serum VEGF-D are higher in patients with a greater extent of cysts as evaluated by CT scan [23, 34]. To the best of our knowledge, this study is the first to show an association of the tissue expression of VEGF-D with pulmonary cystic destruction and loss of pulmonary function in LAM patients.

The 10-year survival of LAM patients varies from 40 to 91% [35, 36] and DL_CO_, FEV_1_, histology score and tomographic changes are considered the main prognostic markers [37]. We observed that, except for D2-40, all immunohistological parameters evaluated were significantly different between the two severity groups, which reinforces their association with functional impairment. Furthermore, the observation that patients with higher tissue expression of mTOR, VEGF-D and MMP-9 presented significantly lower survival or a greater risk to undergo lung transplantation, emphasizes a potential association of the extent of those immunohistological changes with prognosis.

## Conclusions

We conclude that severe LAM patients present with high expression of VEGF-D, mTOR, and MMP-9, as well as cystic destruction and LAM cell infiltration. The tissue expression levels of VEGF-D and MMP-9 are important parameters associated with the loss of pulmonary function and could be considered as potential severity markers in open lung biopsies of LAM patients. Future studies are necessary to establish whether the expression of such tissue markers affects the response to treatment modalities.

## Abbreviations

CT: Computed tomography
D2-40: Podoplanin
DLco: Diffusion capacity for carbon monoxide
FEV_1_: Forced expiratory volume in the first second
FVC: Forced vital capacity
H&E: Hematoxylin and eosin
HMB45: Human melanoma black 45
LAM: Lymphangioleiomyomatosis
MMP-9: Metalloproteinase - 9
mTOR: Mammalian target of rapamycin
PFT: Pulmonary function tests
RV: Residual volume
SMA: Smooth muscle actin
TLC: Total lung capacity
TSC: Tuberous sclerosis complex
VEGF: Vascular endothelial growth factor
VEGFR-3/Flt-4: Vascular endothelial growth factor receptor 3
